# PET/CT imaging of *Mycobacterium tuberculosis* infection

**DOI:** 10.1007/s40336-016-0164-0

**Published:** 2016-03-07

**Authors:** Alfred O. Ankrah, Tjip S. van der Werf, Erik F. J. de Vries, Rudi A. J. O. Dierckx, Mike M. Sathekge, Andor W. J. M. Glaudemans

**Affiliations:** Department of Nuclear Medicine and Molecular Imaging, University Medical Centre Groningen, University of Groningen, Hanzeplein 1, 9700 RB Gronigen, The Netherlands; Department of Internal Medicine, Infectious Diseases, and Pulmonary Diseases and Tuberculosis, University Medical Centre Groningen, University of Groningen, Groningen, The Netherlands; Department of Nuclear Medicine, University of Pretoria, Pretoria, South Africa

**Keywords:** PET/CT, Tuberculosis, 3′-Deoxy-3′-[^18^F]fluoro-l-thymidine, ^18^F-fluoroethylcholine, ^68^Ga-citrate

## Abstract

Tuberculosis has a high morbidity and mortality worldwide. *Mycobacterium tuberculosis* (*Mtb*) has a complex pathophysiology; it is an aerobic bacillus capable of surviving in anaerobic conditions in a latent state for a very long time before reactivation to active disease. In the latent tuberculosis infection, the individual has no clinical evidence of active disease, but exhibits a hypersensitive response to proteins of *Mtb*. Only some 5–10 % of latently infected individuals appear to have reactivation of tuberculosis at any one time point after infection, and neither imaging nor immune tests have been shown to predict tuberculosis reactivation reliably. The complex pathology of the organism provides multiple molecular targets for imaging the infection and targeting therapy. Positron emission tomography (PET) integrated with computer tomography (CT) provides a unique opportunity to noninvasively image the whole body for diagnosing, staging and assessing therapy response in many infectious and inflammatory diseases. PET/CT is a powerful noninvasive tool that can rapidly provide three-dimensional views of disease deep within the body and conduct longitudinal assessment over time in one particular patient. Some PET tracers, such as ^18^F-fluorodeoxyglucose (^18^F-FDG), have been found to be useful in various infectious diseases for detection, assessing disease activity, staging and monitoring response to therapy. This tracer has also been used for imaging tuberculosis. ^18^F-FDG PET relies on the glucose uptake of inflammatory cells as a result of the respiratory burst that occurs with infection. Other PET tracers have also been used to image different aspects of the pathology or microbiology of *Mtb*. The synthesis of the complex cell membrane of the bacilli for example can be imaged with ^11^C-choline or ^18^F-fluoroethylcholine PET/CT while the uptake of amino acids during cell growth can be imaged by 3′-deoxy-3′-[^18^F]fluoro-l-thymidine. PET/CT provides a noninvasive and sensitive method of assessing histopathological information on different aspects of tuberculosis and is already playing a role in the management of tuberculosis. As our understanding of the pathophysiology of tuberculosis increases, the role of PET/CT in the management of this disease would become more important. In this review, we highlight the various tracers that have been used in tuberculosis and explain the underlying mechanisms for their use.

## Introduction

### Epidemiology

Tuberculosis (TB) remains a threat to humans with high mortality, rising incidence of multidrug resistance and HIV co-infection despite the availability of relatively cheap and effective treatment options [1–3]. TB kills 1.5 million people annually and 9.6 million develop the disease annually [2]. Most of these deaths are preventable and therefore the death toll is unacceptably high [4]. It is currently the second highest infective cause of death worldwide, only surpassed by human immunodeficiency virus (HIV). With first cases dating back 9000 years, *Mycobacterium tuberculosis* (*Mtb*), the causative agent of TB, is one of the most successful human pathogens of all time [5]. An increasing proportion of the disease is caused by organisms insensitive to first-line chemotherapeutic agents (multidrug-resistant TB [MDR-TB]), first- and some second-line agents (extensively drug-resistant TB [XDR-TB]) or even all agents (super-extensive drug-resistant TB [SXDR-TB]) [6, 7]. This is a major health-care problem worldwide, since treatment of MDR-TB and even worse XDR-TB is more challenging and expensive than drug-susceptible TB [8, 9]. In 95 % of infected individuals, the pathogen is contained as an asymptomatic latent infection. It has been estimated that a third of the world’s population harbors latent TB [10]. The perilous union of TB with HIV also represents a challenging public health priority as HIV weakens our most effective barrier against TB, our immune system, rendering infected individuals more susceptible to TB [11]. HIV causes a sharp increase in the number of LTBI patients who progress to active disease [12]. HIV co-infection also presents diagnostic challenges, potentially delaying diagnosis and treatment and thereby increasing morbidity and mortality. As a consequence of this syndetic interaction, 1.2 million patients with HIV developed TB and 400,000 people co-infected with TB and HIV died [2]. Despite the enormous burden of TB, current diagnostic methods are woefully inadequate to meet clinical and research needs [13].

### Pathophysiology

*Mtb* is an aerobic, obligate intracellular microorganism that features an unusually complex and thick cell wall. Hallmarks are long-chain fatty acids called mycolic acids that surround the bacterial cytoplasmic membrane. The characteristic features of the *Mtb* include its potential to persist in host cells, slow growth, complex membrane and intracellular pathogenesis [14]. *Mtb* persists in host cells in a dormant, latent or persistent state using a specific genetic program to respond to stress [15, 16]. This program, formerly referred to as Dos Regulon, now DevR activity, is essential for regulon induction and hypoxic survival of *Mtb* [17]. Latency is defined clinically by reactive tuberculin skin test indicating delayed hypersensitivity to *Mtb* antigens in the absence of active disease. Persistence is used to describe to the state in which *Mtb* survives in host tissues under various stress conditions. Dormancy refers to a state in which *Mtb* remains quiescent within infected cells and is the result of metabolic and replicative shutdown of the bacillus using its DevR activity, resulting from the action of a cell-mediated response of the host that can contain but not eradicate the infection [18]. The generation time of actively replicating *Mtb* in synthetic medium or infected animals is about 24 and 18 h in humans [19, 20]. This contributes to the chronic nature of the disease, imposing lengthy treatment regimens and presenting a formidable obstacle for researchers. The slow growth of *Mtb* necessitates long antibiotic therapy rendering treatment susceptible to failure due to non-adherence [21]. The drugs used involve unpleasant side effects, and travel to treatment posts poses economic difficulties to patients. Notably, treatment failure is the major fuel for the development of drug resistance [22]. The mycobacterial cell wall is impermeable to a number of compounds, a feature in part responsible for inherent resistance to numerous drugs [23]. While mycobacteria are considered Gram positive, the second membrane executes biological functions comparable to the outer membrane of Gram-negative bacteria, such as the uptake of small hydrophilic nutrients via special membrane channels [24]. This protective outer membrane plays an important role in securing the bacillus’ integrity in the face of harsh environmental conditions [25]. This outer compartment of the cell wall consists of both lipids and proteins, some of which are linked to polysaccharides. The lipid-linked polysaccharides associated with the outer cell wall consist of lipoarabinomannan (LAM), lipomannan and phthiocerol-containing lipids such as phthiocerol dimycocerosate, dimycolyl trehalose (cord factor), sulfolipids and the phosphatidylinositol mannosides [23]. The pathogenic effects of some of the lipids include the following: LAM inhibits T cell proliferation and has bactericidal action of macrophages amidst other actions. Cord factor, another glycolipid, inhibits phagosome–lysosome fusion, contributing to the maintenance of the granuloma response. It is toxic to macrophages, killing them on contact [26, 27]. The success of *Mtb* as a pathogen lies in its ability to orchestrate its metabolic pathways to survive in a nutrient-deficient, acidic, oxidative, nitrosative and hypoxic environment inside granulomas or infective lesions and survive in its host for months to decades in an asymptomatic state, using DevR activity [28, 29]. The pathogenic potential of *Mtb* also depends largely on the type VII secretion system ESX-1, which is largely responsible for the secretion of early secreted antigenic target (ESAT-6), culture filtrate protein (CPF-10) and several other ESX-1 associated proteins. The ESX-1 governs numerous aspects of interaction between *Mtb* and the host cell. The ESX-1 system possesses membrane-damaging activity, allowing *Mtb* to escape from *Mycobacterium*-containing vacuole into host cell cytosol, where it polymerizes with actin and spreads from cell to cell, particularly in the later stage of the infection [30–32].

### Transmission and disease progression

*Mtb* is transmitted as aerosol generated by the respiratory system, and in 95 % of cases in which the bacilli are inhaled, a primary infection is established [33]. The cell-mediated immunity of the host results in either the clearing of the infection or the restriction of the bacilli inside granulomas giving rise to a latent TB infection (LTBI), defined by no visible symptoms of disease, but dormant and yet alive bacilli in the host. The progress of TB can be stalled at this stage in some cases by isoniazid—or other regimens of preventive therapy [34]. This state might last for the entire life span of the individual or progress to active TB by reactivation of the existing infection with a lifetime risk of 5–10 % [35]. In the presence of HIV, this risk increases with 5 % of LTBI reactivating per year [2]. Reactivation of TB usually occurs at the upper more oxygenated lobe of the lung. This can be cured by treatment. In untreated or poorly treated cases, TB lesions develop within the lung. These lesions include caseous necrosis, fibrosis and cavities. The development of cavities close to airways allows shedding of bacilli into airways and subsequent transmission to other people as aerosol.

### Clinical symptoms and risk factors

The classic features of pulmonary TB include chronic cough, weight loss, fever, night sweats and hemoptysis [36]. The risk for development of active TB disease is governed by exogenous and endogenous factors. Exogenous factors accentuate the progression from exposure to infection. Bacillary load in the sputum of the infected person, duration and proximity to an infectious TB case are key factors. Endogenous factors, on the other hand, lead to the progression from infection to active TB disease [37]. Malnutrition, tobacco smoking and indoor air pollution from solid fuel have been documented to be most important risk factors for TB worldwide, followed by HIV infection, diabetes and excessive alcohol intake [38]. Extrapulmonary TB occurs in 10–42 % of patients. The occurrence of extrapulmonary disease depends on the age, presence or absence of underlying disease, ethnic background, immune status of the individual and the strain or lineage of *Mtb* [37]. The disease may occur in any part of the body and can mimic a lot of clinical diseases, which potentially delays the diagnosis. HIV co-infection with TB presents major challenges to the diagnosis and treatment of TB. The manifestation of TB varies depending on the immune status of the host. Soon after HIV infection, TB presentation is similar to HIV seronegative individuals. As the CD4 count drops, the presentation becomes atypical, with atypical pulmonary manifestations and a greater proportion of patients (more than 50 % in some cases) presenting with extrapulmonary disease. At very low CD4 counts, the pulmonary features of disease may be completely absent and disseminated TB may present as a nonspecific febrile illness with high mortality, in which clinical diagnosis may be completely missed and will only be discovered at autopsy [39–42].

## Diagnosis of tuberculosis

Robert Koch first used sputum microscopy and culture to identify TB over 130 years ago. The diagnosis of active TB in many parts of the world has still remained the same [43]. Although inexpensive and accessible, the technique is operator dependent and has a poor sensitivity (45–80 %). Sputum culture has a specificity of 98 %, but it takes 2–8 weeks for results to be available depending on culture media and bacillary burden [44]. Furthermore, sputum is difficult to collect from infants and children and the sensitivity of microscopy of direct acid- and alcohol-fast stains is low, often requiring multiple samples from an individual before the diagnosis can be established [45, 46]. Sputum culture is considered the golden standard for the diagnosis; liquid media have largely replaced solid culture media, e.g., the Lowenstein–Jensen slope; liquid media have been shown to considerably improve sensitivity and speed in diagnosis of active TB [47]. The diagnosis of LTBI differs from active TB. LTBI by definition refers to a clinical state where one has *Mtb* infection without evidence of disease. It is diagnosed by positive immunological response to proteins of *Mtb* in the absence of clinical or radiological findings. Traditionally, the Mantoux tuberculin skin test (TST) has been used to assess the immunological response. This test is conducted by injecting 0.1 ml of a tuberculin-purified protein derivative by the intradermal route on the inner surface of the forearm. This should produce a discrete pale elevation of the skin of about 6–10 mm when done correctly. The test is read by measuring the diameter of the induration of the skin produced. The test is interpreted along with the clinical risk of the individual. While an induration of more than 15 mm is positive, in a person with high risk for disease such as contact with a TB patient a diameter of 10 mm is considered positive. The test has limitations of being falsely negative in particularly immunocompromised patients, while it gives false-positive results in other patients with other mycobacteriosis or people who have had previous vaccinations against TB [47].

### Imaging in response to diagnostic limitations

Plain chest radiography (and in affluent settings, also CT) are the mainstays for diagnostic imaging of pulmonary TB, but are often nonspecific and unable to provide a definitive diagnosis due to the heterogeneous presentation, particularly in case of HIV co-infection when CD4 counts are low [48–50]. On radiographs, primary TB is represented by consolidation (Ghon focus), adenopathy and pleural effusion. The Ghon focus most commonly occurs in the mid and lower lung zones. When there is hilar lymphadenopathy in addition to the Ghon focus, a Ghon complex is formed. The radiological features of reactivation TB include focal patchy opacities, cavitation, fibrosis, nodal calcification and flecks of caseous material. These commonly occur in the posterior segments of the upper lobes and superior segment of the lower lobes of the lungs. These features may be completely absent in patients with severe immune deficiency. As a result of the limitations of traditional diagnostics, new approaches have been developed. The interferon gamma release assay (IGRA) is a blood test measuring cellular immune response to the TB infection similar to the traditional tuberculin skin test (TST), with similar sensitivity and improved specificity in BCG-vaccinated individuals [51]. However, neither IGRA nor TST is able to distinguish latent infection and active disease and they are both dependent on host responses, which may be compromised in immune-deficient patients and children [52, 53]. Other new diagnostic methods include urine LAM testing, which detects active TB in HIV patients with high specificity, but modest sensitivity [54]. The Xpert MTB/RIF assay rapidly detects *Mtb* nucleic acid in sputum, by targeting the rpoB-gene of *Mtb*, and at the same time genetic mutations predicting rifampicin resistance. It has a specificity of 98 %, but variable sensitivity depending on the sputum bacterial load [55, 56]. Albeit being useful for rapid initial diagnosis, it only establishes susceptibility to rifampin and is inferior to culture for monitoring treatment [57]. During treatment, subsets of *Mtb* within the bacterial population that reflect naturally occurring drug-resistant mutants may emerge. This usually occurs during sub-optimal treatment with inadvertent monotherapy. Most currently used diagnostic systems including liquid culture fail to detect such subsets at the start of treatment. Currently used diagnostic platforms accept that if <1 % of the bacterial population is resistant to a particular drug at one particular drug concentration (the so-called breakpoint) that is generally accepted to be achieved in blood by using the standard TB dosing regimen, that strain of *Mtb* should be considered susceptible to that drug. The idea behind accepting *Mtb* at that breakpoint is the assumption that by always using a regimen containing three to five active drugs, the treatment will not fail because the <1 % *Mtb* resistant to a particular drug will be covered by other components of the drug regimen. Current breakpoints recommended by EUCAST have recently been questioned. In vitro hollow fibber models mimicking plasma-drug concentration and population kinetics demonstrated that an important subset of patients might not reach target drug concentrations over time. These individuals may fail on regimes that according to the currently used EUCAST breakpoint would have drug-susceptible TB. Furthermore, very few of the currently available drugs have efficacy in slowly replicating persistent organisms [58, 59]. PET/CT may provide an important tool in the detection of this subset of *Mtb* population.

### ^18^F-FDG PET

**Fig. 1:**
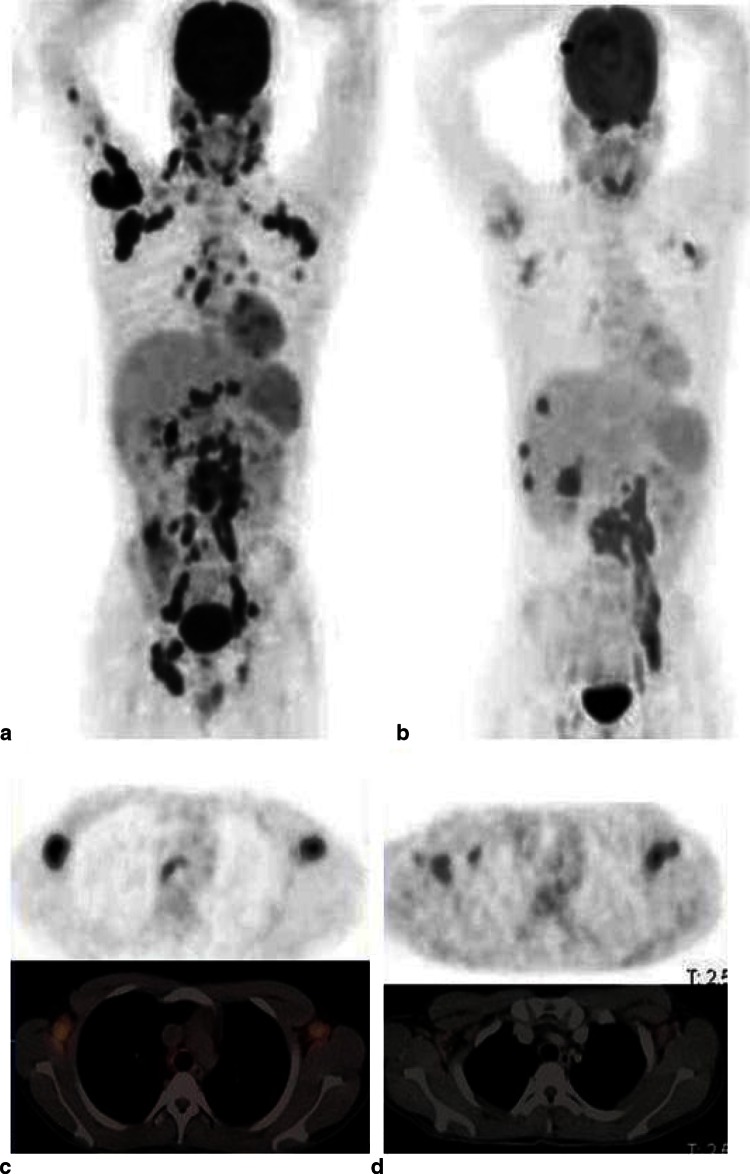
^18^F-FDG PET/CT scan before anti-TB treatment and 2 months after initiation of treatment for interim assessment of treatment response. **a** Maximum intensity projection (MIP) image before treatment (PET images only), showing extensive disease: pulmonary, cervical, axillary, mediastinal, abdominal, pelvic and inguinal lymph nodes, hepatic and skeletal metastasis to the lumbar spine and right humerus. **b** MIP image after 2 months of anti-TB treatment (PET images only): complete metabolic response of the pulmonary and right humeral lesions and the pelvic and inguinal lymph nodes. Good metabolic response in the mediastinal, cervical and axillary nodes. Active disease is still present in the lumber spine with progression of the hepatic lesions. **c** Transverse scans showing axillary nodes before treatment (PET and integrated PET/CT images). **d** Transverse scans showing response of axillary nodes after 2 month of anti-TB therapy; nodal uptake diminished but still present

PET/CT has the ability to associate the pharmacological, immunologic and microbiological aspects of TB lesions with anatomic information, allowing a holistic approach to understanding the disease [14]. The value of imaging TB with 2-[^18^F]fluoro-2-deoxyglucose (^18^F-FDG) PET/CT has been well documented [60–62]. ^18^F-FDG PET has been used to detect TB granulomas and assess disease activity [63, 64] and the extent of disease [65]. Efforts to use ^18^F-FDG PET to distinguish benign from malignant lesions have been made and results have generally not been encouraging [66, 67]. Active TB avidly takes up ^18^F-FDG, both in pulmonary and extrapulmonary lesions. Thus, ^18^F-FDG PET can be very useful to assess the extent of active TB. The detection of extrapulmonary lesions is particularly useful, as obtaining tissue or fluid for analysis may not always be possible or may be invasive. Many studies have been conducted to distinguish avid TB from malignancy and other granulomatous conditions [68–71]. Since ^18^F-FDG is a nonspecific tracer, it cannot reliably distinguish tuberculomas from malignant lung lesions and frequently gives rise to a false-positive diagnosis in patients evaluated for malignancy [72–74]. The overlap between the standardized uptake value (SUV) in malignant and benign lesions has led to the investigation of several dichotomization methods, such as the use of SUV cutoff thresholds, dual tracer imaging, dual time point imaging (DTPI) or delayed imaging. There is, however, no consensus about the use of ^18^F-FDG PET to differentiate TB from malignancy or other granulomatous or other inflammatory lesions. In a review, Cheng et al. point out that the increased specificity of dual-time point imaging depends on several factors and therefore recommend the selective use of DTPI to improve the diagnostic accuracy and interpretation confidence only in specific situations These situations include obese, overweight or poorly controlled diabetic patients who have high background uptake of ^18^F-FDG [75]. It is important for the interpretation of an ^18^F-FDG PET scan to have a high suspicion for TB, particularly in a TB-endemic area or for an immigrant from an endemic area now living in an area with low TB prevalence [76]. Other studies have evaluated the use of ^18^F-FDG PET in differentiating latent from active TB [77, 78]. TB has recently been shown to be more dynamic than previously thought [79, 80]. The infection occupies a more diverse spectrum rather than simply latent and active disease (Fig. 1). Thus, while high uptake in a lesion in a patient with TB may represent active disease, it may also represent a host immune system response that will eventually prevail [78]. It is therefore prudent to exercise when interpreting high 18F-FDG uptake as active TB in a patient with no known history of active disease or symptoms of TB, but only a positive tuberculin skin test or interferon gamma release assay. ^18^F-FDG PET has also been used to evaluate treatment response during and after therapy (Fig. 1). Treatment of TB is a lengthy process, usually taking at least 6 months, and resistant *Mtb* species are emerging. It is important to have an early test to predict outcome early in the course of treatment to enable timely change to appropriate therapy to prevent resistant species. ^18^F-FDG PET has been shown to be very useful in monitoring treatment response in this regard [81, 82]. Other studies also used ^18^F-FDG PET to assess response on completion of treatment and some studies, in particular in the context of MDR-TB, have shown patients remaining free of TB months after completing treatment [83–85]. ^18^F-FDG has also been evaluated and found to be useful for assessing TB in specific organs of the body, such as tuberculous infections of the skeleton. It has been shown to help in distinguishing acute pyogenic from chronic tuberculous spondylitis [86–88]. ^18^F-FDG PET has, however, not been useful in distinguishing TB from atypical TB, sarcoidosis and HIV-associated lymphadenopathy [89–91]. An overview of original publications involving ^18^F-FDG PET in more than one patient with TB is presented in Table 1. The articles were found by entering PET/CT and tuberculosis in medical Pubmed and all the references of those articles were reviewed for additional references. Publications including only one case were excluded.

**Table 1:** Original articles on the use of ^18^F-FDG PET in TB

Journal/year	1st author	Use	No. of TB pts/pts studied	Major finding of ^18^F-FDG and TB	Sens (%)	Spec (%)
Ann Nuc Med 1996	Ichiya et al. [63]	1, 6	8/24	Detected and assessed activity in TB lesions, but was unable to distinguish TB from MAC*	na	Na
Radiology 2000	Goo et al. [64]	1, 3	10/10	Active tuberculomas were ^18^F-FDG avid and caused false positives in cancer evaluation	na	Na
Chest 2003	Hara et al. [66]	1, 3, 6	14/116	TB, atypical TB and cancer were discriminated by performing both ^18^F-FDG and ^11^C-choline PET scans	na	Na
Neoplasia 2005	Mamede et al. [67]	1, ***3***	10/60	Uptake correlated with inflammation of TB lesions causing false-positive results in cancer	87–97.8	Na
Tuberculosis 2007	Hofmeyr et al. [83]	3, 5	2/2	Was useful in TB diagnosis in high-risk patients and in monitoring anti-TB treatment	na	Na
Clin Nuc Med 2008	Park et al. [84]	5	2/2	Was useful in assessing response to anti-TB therapy in patients with tuberculoma	na	Na
EJNMMI 2008	Yen et al. [68]	3	8/96	TB was a major cause of false positives in evaluating lymph nodes in lung cancer	73.8	88.9
EJNMMI 2008	Kim et al. [75]	4	25/25	Assessed TB activity by visual assessment and SUV change from early to delayed scan	71.4–100	81.8–100
EJNMMI 2009	Demura et al. [81]	1, 4, 5	25/47	Distinguished latent TB from active TB and in monitoring anti-TB therapy response	na	Na
Nuc Med Comun 2009	Castaigne et al. [103]	1, 6	6/10	Was useful in detecting TB as a cause of fever of unknown origin in HIV patients	na	Na
Pediatr Surg Int 2009	Hadley et al. [68]	3, 6	3/18	Was a major cause of false positive for cancer in HIV children	na	Na
Spine 2009	Kim et al. [86]	5, ***6***	11/30	Had prognostic value in anti-TB therapy of the spine and detected residual disease	85.7–100	68–82.6
Lung 2010	Hahm et al. [90]	1, 6	26/41	Was unable to distinguish TB from MAC	na	Na
World J Gastroenterol 2010	Tian et al. [70]	3	3/3	Was a cause of false positive in assessing abdominal malignancy	na	Na
Nuklearmedizin 2010	Sathekge et al. [65]	2, 7	16/16	Detected more extensive disease when compared to contrast-enhanced CT	na	Na
Acta Radiol 2010	Tian et al. [92]	5	3/3	Was useful in assessing response to treatment in non-pulmonary TB	na	Na
S Afr Med J 2010	Sathekge et al. [93]	3	12/30	Was not useful in differentiating benign from malignant lesions in a TB-endemic area	87	25–100
QJNMMI 2010	Sathekge et al. [89]	3, 6	37/83	Was not useful for assessing malignancy in lymph nodes in TB, HIV or TB and HIV co-infection	Na	Na
Nuc Med Commun 2011	Kim et al. [87]	6	8/23	Was useful in distinguishing TB spondylitis from pyogenic spondylitis	86.6	62.9
Ann Nuc Med 2011	Li et al. [94]	3	8/96	TB caused high false positives for cancer with PET only; accuracy improved with combined PET/CT	96.7	75.7
Ann Thoracic Med 2011	Kumar et al. [95]	1, ***3***	12/35	Increased SUV cutoff improved specificity and with acceptable sensitivity in mediastinal node evaluation	87–93	40–70
J Korean Med Sci 2011	Lee et al. [71]	***3***, 6	54/54	Found low accuracy in the evaluation of lung cancer pts with parenchymal sequelae from previous TB	60	69.2
J Nucl Med 2011	Sathekge et al. [82]	1, 2, ***5***	24/24	Was useful to predict HIV patients who would respond to anti-TB therapy	88	81
Eur J Rad 2012	Soussan et al. [96]	2	16/16	Found 2 distinct patterns of pulm TB uptake	na	Na
EJNMMI 2012	Sathekge et al. [97]	***5***, 7	20/20	Was useful in distinguishing lymph nodes responding to anti-TB from those that did not	88–95	66–85
Int J Tuberc Lung dis 2012	Martinez et al. [98]	5	21/21	Was useful in evaluating early therapeutic response to anti-TB	na	Na
BMC Pulm Med 2013	Heysell et al. [78]	2, 4	4/4	Demonstrated the usefulness in the management of high-risk TB pts who are sputum negative	na	Na
Eur Spine J 2014	Dureja et al. [88]	5	33/33	SUVmax was found to be a quantitative marker for response in spinal TB	na	Na
Sci Trans Med 2014	Coleman et al. [99]	5	18/18	Demonstrated usefulness of assessing the response of anti-TB in macaques and pts with XDR-TB	96	75
J Korean Med Sci 2014	Jeong Y-J et al. [100]	1	63/63	Found pts with old healed lesions with high SUV to be at risk for development of active TB	na	Na
Sci Trans Med 2014	Chen et al. [85]	5	28/28	Demonstrated that changes at 2 months of anti-TB are early predictors of the final outcome in MDR-TB	na	Na
Chest 2014	Maturu et al. [91]	6	29/117	Did not find any significant difference in the findings in TB and sarcoidosis	na	Na
Nuc Med Commun 2015	Huber et al. [101]	3, 6	122/207	Found more likely to detect cancer in the evaluation of granulomatous lesions in pts > 60 years	na	Na
EJNMMI 2015	Fuster D et al. [102]	7	4/26	Recommended ^18^F-FDG should be considered first line in the imaging of spondylodiscitis	83	88

When an article evaluated more than one feature of TB, then the sensitivity and specificity apply to the use indicated by the number highlighted in italics and bold

*Pts* patients, *MAC* *Mycobacterium avium* complex

* *Pulm* pulmonary

^1^To detect TB lesions and assess disease activity

^2^To assess the extent of disease

^3^To assess the effect of TB on cancer staging or diagnosis with ^18^F-FDG

^4^To differentiate latent from active TB

^5^To monitor treatment response

^6^To assess the ability to differentiate TB from nonmalignant conditions, including atypical mycobacteria, as well as to assess the effect TB has on ^18^F-FDG imaging of nonmalignant conditions

^7^To compare the detection of TB by PET with other modalities

### Other PET tracers

Besides ^18^F-FDG, other PET tracers have been investigated for imaging of TB, including ^11^C-choline, [^18^F]fluoroethylcholine (^18^F-FEC), 3′-deoxy-3′-[^18^F]fluoro-l-thymidine (^18^F-FLT), ^68^Ga-citrate, [^18^F] sodium fluoride (^18^F-NaF) and radiolabeled anti-TB drugs (Table 2). The wall of *Mtb* consists of many complex lipids. ^11^C-choline or ^18^F-FEC can image the transportation and utilization of choline in this lipid-rich envelope of *Mtb*. The incorporation of thymidine into the DNA of bacteria can be imaged by the thymidine analog ^18^F-FLT during the proliferation of *Mtb*. The uptake of ^68^Ga-citrate by TB can be extrapolated from studies with ^67^Ga-citrate and is dependent on specific and nonspecific uptake mechanisms. Nonspecific mechanisms include increased vascular permeability in areas of inflammation, while specific factors include binding to the siderophores the bacteria used to trap iron from hosts’ transferrin and other iron sources. ^18^F-NaF has a strong binding affinity for calcium and can be used to visualize micro-calcifications in old TB lesions. Drugs used for the treatment of TB have also been labeled with radioisotopes and can be used to study the biodistribution and pharmacokinetics of these drugs.

**Table 2:** Mechanism of PET tracer uptake in TB

Tracer	Clinical or pre-clinical (animal model used)	Mechanism of uptake	Use(s)
^18^F-fluoro-deoxy-glucose [60–103]	Clinical	Uptake during the respiratory burst by activated inflammatory cells as by glucose transporters and is phosphorylated to FDG-6-phospate and remains trapped in the cell	Assesses disease activity, staging (especially extrapulmonary) monitoring therapy and early prediction of nonresponse
^18^F-Fluoroethylcholine or ^11^C-choline [104–106]	Clinical	Uptake during the synthesis of the complex lipid layer of the cell wall	Combined with FDG, helps distinguish TB from malignancy and possible role in therapy monitoring
3′-Deoxy-3′-^18^F-fluoro-l-thymidine [107, 108]	Clinical	Uptake during the synthesis of nucleic acids as bacteria proliferates	Combined with FDG, helps distinguish TB from malignancy
^68^Ga-citrate [109, 110]	Clinical	Accumulates in bacterial siderophores of *Mtb* and in plasma lactoferrin similar to ^67^Ga-citrate and also accumulates by nonspecific mechanisms as increased vascular permeability	Detects TB lesions and may be better than CT in the detection of extrapulmonary lesions
^11^F-sodium fluoride [111]	Preclinical (mice)	Binds to micro-calcification in chronic TB lesions	Potentially helps to distinguish acute from chronic TB
^11^C-Rifampicin [112]	Preclinical (baboons)	Binds to (and inhibits) *Mtbs* DNA-dependent RNA polymerase	Determines whether there is adequate accumulation of drug in the infected site
^11^C-Isoniazid [112]	Preclinical (baboons)	Binds to *Mtb* enzymes and generates reactive oxygen species resulting in inhibition of cell wall lipid synthesis and depletion of nucleic acid pools and metabolic depression	Determines whether there is adequate accumulation of drug in the infected site
^11^C-Pyrazinamide [112]	Preclinical (baboons)	Binds to cell membrane proteins, disrupts membrane energetics and inhibits membrane transport functions in *Mtb*	Determines whether there is adequate accumulation of drug in the infected site

#### ^11^C-Choline/^18^F-fluoroethylcholine

^11^C-Choline was evaluated for differentiation of lung cancer and other lesions including active TB [66, 104, 105]. While 75 % accuracy was found when using ^11^C-choline alone for this differentiation, combining ^11^C-choline and F-FDG appeared to yield better results [105]. Both tracers displayed a high uptake in malignant lesions. In TB, however, ^18^F-FDG uptake was much higher than ^11^C-choline uptake. The use of only one tracer may miss extrapulmonary disease in areas where the tracers have a physiologically high bio-distribution such as the brain for ^18^F-FDG and the liver for ^18^F-FEC [106]. ^18^F-FEC has been suggested to be useful for evaluation of TB therapy.

#### 3′-Deoxy-3′-^18^F-fluoro-l-thymidine

Prospective studies evaluating the diagnostic value of dual tracer PET/CT in pulmonary lesions using ^18^F-FLT and ^18^F-FDG PET noted that ^18^F-FLT PET is most useful when combined with ^18^F-FDG PET. This yielded more information than either tracer used alone. Visual inspection of images and the ratio of the maximum SUV between ^18^F-FLT and ^18^F-FDG improved the diagnostic accuracy in distinguishing malignant from benign lesions including tuberculosis [107, 108].

#### ^68^Ga-citrate

^68^Ga-citrate was shown to have good uptake in both pulmonary and extrapulmonary TB lesions and was useful in distinguishing active lesions from inactive lesions. However, the uptake was nonspecific, as it was unable to distinguish malignant from benign lesions [109, 110]. ^68^Ga-citrate is potentially a very useful tracer to stage disease in cases where the diagnosis is already known. Further studies are needed to see if ^68^Ga-citrate will have the same use as demonstrated by ^18^F-FDG (Table 1). ^68^Ga-citrate holds promise particularly in middle income and or even developing economies where PET/CT may be available, because ^68^Ga is produced from a generator rather than from a cyclotron. The expense and high technical demands for running a cyclotron have been obviated and the tracer will be readily available.

#### ^18^F-sodium fluoride

A study demonstrated in a murine model of chronic TB the usefulness of ^18^F-NaF in detecting micro-calcifications, which were not visualized by CT. This approach could potentially be applied in humans and help distinguish acute from chronic TB [111].

#### ^11^C-radiolabeled drug tracers

Some chemotherapeutic agents for treatment of tuberculosis, including isoniazid, rifampicin and pyrazinamide, have been labeled with carbon-11 (^11^C) and their biodistribution, in particular their ability to cross the blood–brain barrier, has been evaluated in nonhuman primates. There have not been any corresponding human studies with these tracers till date. The radiolabeled chemotherapeutic agents were used to show whether the drug achieved a sufficiently high concentration in infected sites, particularly in TB meningitis and TB brain abscesses. This is an important finding, as TB of the central nervous system is usually life threatening and requires long periods of treatment (usually a year) and may sometimes need to be continued even when adverse effects develop. These radiolabeled drug tracers were not assessed for detection of TB [112].

### Treatment of TB

The treatment of TB depends on whether the individual has active or LTBI. Treatment of active TB requires long-term multidrug therapy to overcome tolerance, achieve bacterial clearance and reduce the risk of transmission. Tolerance is an epigenetic drug resistance widely attributed to nonreplicating bacterial subpopulations [113]. The drugs are classified as first- and second-line agents. First-line agents—class 1, WHO—include isoniazid and rifampicin, the two most potent anti-TB agents. Resistance to these agents defines a case of MDR-TB. Second-line agents are divided by the WHO into four different classes (WHO class 2–5); class 2 includes the injectables amikacin, kanamycin and capreomycin; class 3 are the fluoroquinolones; class four includes less potent, more toxic oral agents; and class 5 includes agents with as yet unknown significance. Only if *Mtb* isolates are susceptible, the treatment may last 6 months. Patients with large cavitary lesions that have delayed sputum culture conversion, those with *M. bovis* disease that is naturally nonsusceptible to pyrazinamide, those who cannot tolerate pyrazinamide and those with meningitis TB need treatment prolongation to 9 months. The so-called “short course” of 6 months for drug-susceptible TB is a major advance, as previous therapies lasted 12–18 months. Treatment is still challenging, as adhering to a multidrug regimen for 6 months has been shown to be difficult, especially in low-resource settings [114]. Despite the presence of several new drugs under investigation, attempts to shorten the treatment still remain elusive [115]. This failure highlights the poor understanding of the tolerance of *Mtb*. The WHO recommends isoniazid and rifampicin for treatment of drug-susceptible infections. The treatment is in two phases: initiation and continuation. The initiation phase treatment usually contains four first-line agents including rifampicin and isoniazid for 2 months, and the continuation phase consists of isoniazid and rifampicin only for the last 4 months of treatment. The current guidelines recommend the directly observed treatment (DOTS) strategy to improve adherence. This strategy involves patients taking medication under supervision. Although this has greatly improved the success of treatment, the emergence of drug resistance could not be curbed with this strategy. There are also guidelines for drug-susceptibility testing to rapidly diagnose and appropriately treat MDR-TB [116]. Although the treatment outcome of drug-susceptible TB has been fair, with successful outcome reported by most countries at around 85 %, the outcome of MDR-TB treatment has generally been poor, with successful outcome reported under service conditions at around 50 % only [2]. The reported series do slightly better at around 60 % [117] and only few national programs attain success rates at around the target set for drug-susceptible TB [118].

In patients with LTBI, treatment is recommended for persons deemed to be at high risk of developing active disease. It is important that treatment is only initiated after active disease has been excluded by clinical and radiographic means. A failure to do so will result in inadequate treatment and development of resistant species. The preferred treatment is isoniazid daily for 9 months. The WHO recently developed the guidelines for latent TB treatment. The identification of people at risk considers factors such as the level of income of a country, prevalence of TB in a country, presence of other diseases, conditions like HIV infection and use of gamma interferon. Other guidelines combine these conditions with the size of induration after a tuberculin skin test [2, 119, 120].

## Conclusions and future perspectives

^18^F-FDG PET/CT is a sensitive noninvasive biomarker for the detection, staging, assessing disease activity and monitoring therapy in TB. The complex and long period required for the treatment of TB makes ^18^F-FDG PET particularly useful, as it is able to detect at an early point in treatment drug combinations that are ineffective and lead to a change in therapy. This is not only important to reduce morbidity and mortality in the individual, but prevents the even greater public health hazard of the individual developing resistant species of *Mtb* and transmitting the resistant strain in the community.

PET/CT provides a unique opportunity for the in vivo histological characterization of TB lesions. This role is becoming more and more important as the molecular basis of TB is elucidated. These tracers provide an ideal platform for personalized medicine in TB treatment. PET/CT has played and continues to play a major role in the development of new drugs and therapeutic strategies like vaccines. This role will hopefully expand in the future with the development of new tracers and repurposing of existing tracers. For example, MDR-TB and LTBI have hypoxia as one of the main processes underlying their pathology. The antibiotic metronidazole that is a pro-drug activated by hypoxia has been shown to be useful in the treatment of MDR-TB [121]. Hypoxic PET tracers already validated for the management of cancer could potentially play a role in the management of TB. In conclusion, PET/CT has demonstrated its usefulness in managing different aspects of tuberculosis disease and the development of new therapeutic interventions. The role of PET/CT is likely to grow further as we aim to eradicate TB by 2015 [2].
